# Transcriptional activation of *BolBCAT4* genes enhanced aliphatic glucosinolate accumulation in cabbage

**DOI:** 10.3389/fpls.2025.1548003

**Published:** 2025-04-15

**Authors:** Chengtai Yan, Jiahao Zhang, Wenjing Yang, Yao Liu, Xue Bai, Qi Zeng, Xifan Liu, Yanjing Ren, Dengkui Shao, Baohua Li

**Affiliations:** ^1^ State Key Laboratory for Crop Stress Resistance and High-Efficiency Production, College of Horticulture, Northwest A & F University, Yangling, Shaanxi, China; ^2^ Academy of Agriculture and Forestry Sciences, Qinghai University, Xining, China; ^3^ Laboratory of Research and Utilization of Germplasm Resources in Qinghai-Tibet Plateau, Qinghai University, Xining, China; ^4^ Qinghai Key Laboratory of Vegetable Genetics and Physiology, Xining, China

**Keywords:** cabbage, aliphatic glucosinolates, BolBCAT4, BolMYB3R, transcriptional regulation

## Abstract

Aliphatic glucosinolates are a large group of plant-specialized metabolites in *Brassica* vegetables, and some of their degradation products are key nutrients with significant beneficial effects on human health. Increasing the nutritional quality is one of the central research questions and breeding goals for *Brassica* vegetables. Major progress has been made in understanding transcriptional regulation of aliphatic glucosinolates biosynthesis in the model plant, while little is known about it in *Brassica* vegetables. In this study, we used cabbage to study the transcriptional regulation of *BolBCAT4* genes, the first set of biosynthetic genes in methionine-derived aliphatic glucosinolate metabolism, and identified and functionally validated four upstream positive regulators of *BolBCAT4* genes, BolMYB3R, BolbHLH153, BolMED4 and BolERF74, with consistent phenotypic effects of inducing short-chain aliphatic glucosinolates, including glucoraphanin. Our work confirmed the biological functions of *BolBCAT4* genes, identified dozens of candidate upstream regulators, and provided valuable regulatory mechanisms and breeding targets for enhancing the nutritional quality of cabbage.

## 1 Introduction

Plants produce huge numbers of small molecular metabolites, generally known as plant secondary metabolites, and their structural and functional diversities play key roles in supporting human society, serving as indispensable nutrients, key components in functional foods, and potential natural medicines, with primary metabolites as their precursors in most cases (Burow et al., 2010; Erb and Kliebenstein, 2020; Wink, 1988). The accumulation of plant secondary metabolites is under complex genetic regulation, and highly responsive to the growth conditions and surrounding environments (Kliebenstein, 2023). Key transcriptional factors were identified and showed to turn on and off the whole plant secondary metabolism pathways, including cucurbitacin C in cucurbits (Shang et al., 2014), cyanogenic diglucoside amygdalin in almond (Sanchez-Perez et al., 2019), and essential oils in citrus (Wang et al., 2024). As many of these plant secondary metabolites are highly valuable nutrients or raw materials for producing medicine and they are present in specific tissues at relatively low concentrations, there is high potential for translational applications of plant secondary metabolism to make transcriptional regulation a key research area.

Glucosinolates are a large group of plant secondary compounds containing sulfur and nitrogen. They exist almost exclusively in cruciferous plants and can be categorized as aliphatic, indolic, and benzenic glucosinolates by their biosynthetic precursors (Halkier and Gershenzon, 2006; Sønderby et al., 2010). Aliphatic glucosinolates are the most diverse and biologically rich, mainly due to six elongation steps for methionine-derived biosynthetic products. Many of the key understandings and breakthroughs in studying transcriptional regulation of plant secondary metabolism resulted from research in the area of aliphatic glucosinolates, likely due to the fact that *Arabidopsis* is the model plant and it contains highly diverse aliphatic glucosinolates (Kliebenstein et al., 2001). In 2007, MYB28, MYB29, and MYB76 were identified as the key regulators of aliphatic glucosinolates, and importantly, no aliphatic glucosinolates were detected in the *myb28/myb29* double mutant (Gigolashvili et al., 2007; Hirai et al., 2007; Sønderby et al., 2007). In 2013, in the study of the JA signaling pathway, MYC2, MYC3, and MYC4 were identified as key regulators of both aliphatic and indolic glucosinolates pathways, and no glucosinolates were detected in *myc2/3/4* triple mutant (Schweizer et al., 2013). Starting in 2014, we have systematically screened and functionally validated the transcriptional regulators of the aliphatic glucosinolates pathway in model plants by identifying more than 60 novel regulators and revealing how these regulators interact to regulate the biosynthesis of glucosinolates, connect to plant primary metabolism, and coordinate plant defenses and plant development in *Arabidopsis* (Chen et al., 2024; Li et al., 2014a, 2020, 2018; Tang et al., 2021). Although major advances have been made in model plants, little is known about the transcriptional regulation of aliphatic glucosinolates in *Brassica* vegetables.

*Brassica* vegetables play key roles in providing nutrients in the human diet, and glucosinolates are one of their core nutritional components, providing the unique flavor of cruciferous vegetables, especially the spicy taste (Fahey et al., 2001). Among all the glucosinolates, sulforaphane (SFN) derived from 4-methylsulfinylbutyl glucosinolate (4MSO, also known as glucoraphanin) is the most prominent functional nutrient in *Brassica* vegetables with significance in the suppression of cancers (Liou et al., 2020), including lung cancer, breast cancer, pancreatic cancer, colon cancer, leukemia, and prostate cancer (Aumeeruddy and Mahomoodally, 2019; Elkashty and Tran, 2021; Iahtisham-Ul-Haq et al., 2022; Wu et al., 2020; Yin et al., 2019). Increasing the accumulation of 4MSO is one of the key breeding goals in *Brassica* vegetables and manipulating the transcriptional regulators proved to be a key tool for modulating the accumulation of aliphatic glucosinolates (Neequaye et al., 2021). An understanding of the transcriptional regulatory mechanisms of *Brassica* vegetables is the foundation to achieve this goal.

**Figure 1:**
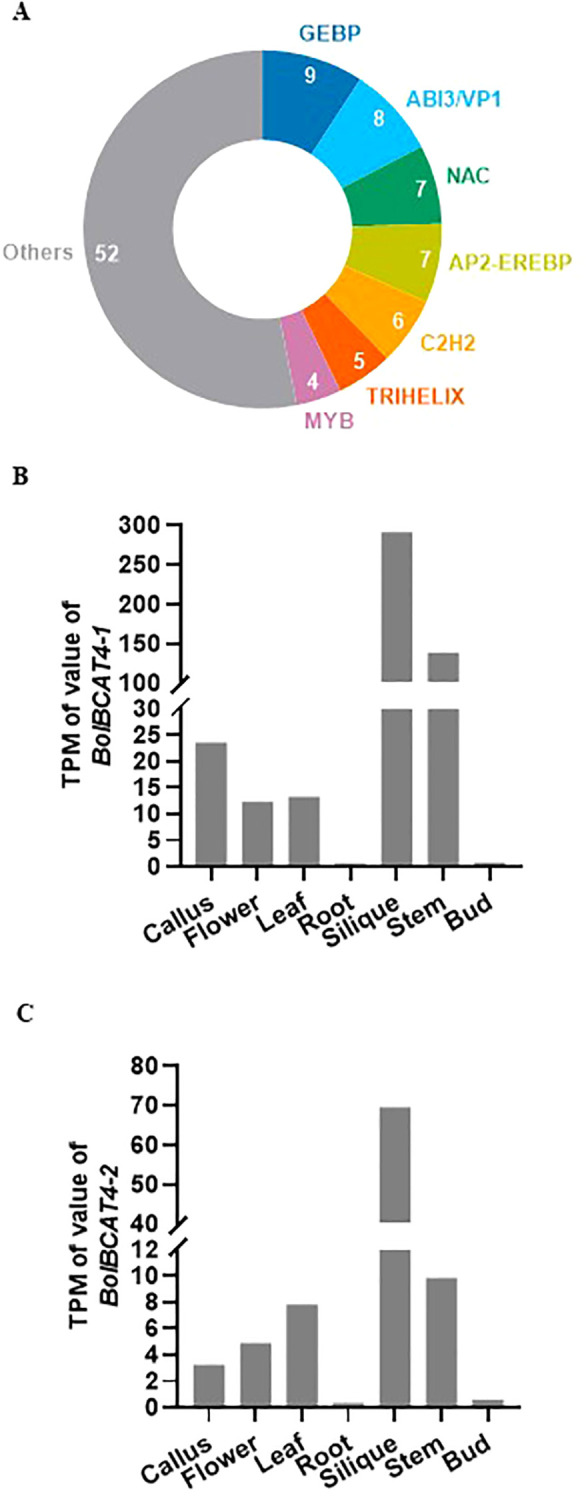
The expression and transcriptional regulation of *BCAT4* genes. **(A)** Distribution of the families of transcription factors binding to the promoter of *AtBCAT4.* **(B)** Expression level of *BolBCAT4-1* gene in representative tissues of cabbage. **(C)** Expression level of the *BolBCAT4-2* gene in representative tissues of cabbage.

The *BRANCHED-CHAIN AMINOTRANSFERASE* (*BCAT*) genes belong to a small gene family, and they function in the branched-chain amino acid metabolic steps; importantly, *BCAT4* was identified as the first biosynthetic gene in the Met-derived aliphatic glucosinolate pathway in *Arabidopsis*, and it converts Met to 2-oxo acid, connects the primary metabolism with the secondary metabolism, and plays key roles in the biosynthesis of aliphatic glucosinolates (Schuster et al., 2006). In addition, *BCAT4* was experimentally found to be strongly suppressed in both the *myb28/myb29* double mutant and the *myc2/myc3/myc4* triple mutant at the transcriptional level, indicating the importance of transcriptional regulation of *BCAT4* in modulating the production of aliphatic glucosinolates (Li et al., 2014a). By using an enhanced yeast one-hybrid assay (Gaudinier et al., 2011), 98 transcriptional factors were identified to bind the promoter of *BCAT4* in *Arabidopsis* (Li et al., 2014a; Tang et al., 2021) (**Supplementary Table 1**), and they belonged to dozens of TF families with diverse biological functions, including MYB, NAC, and C2H2 (**Figure 1A**), indicating these candidate upstream regulators of *BCAT4* coordinate the transcriptional regulation of aliphatic glucosinolates biosynthesis and many diverse biological functions in plants. In this study, we screened, identified, and functionally validated the upstream transcriptional regulators of *BolBCAT4* in cabbage, an important *Brassica* vegetable worldwide. Our study was the first effort thus far to identify the upstream regulators of the biosynthetic genes of the glucosinolate pathway in cabbage, and provided important insights and target genes for breeding new cabbage varieties with higher nutritional value.

## 2 Results

### 2.1 Functional validation of *BolBCAT4* genes in cabbage

To study the transcriptional regulation of *BCAT4* in *Brassica* vegetables, we used cabbage as the experimental material in this study. Due to genomic polyploidization (Liu et al., 2014), two *BCAT4* homolog genes were retrieved from the cabbage genome and renamed *BolBCAT4-1* (*BolC05g044530.2J*) and *BolBCAT4-2* (*BolC03g046400.2J*). The expression patterns of *BolBCAT4-1* and *BolBCAT4-2* showed high expression in the silique and stem, with low expression in roots and buds (**Figures 1B, C**).

**Figure 2:**
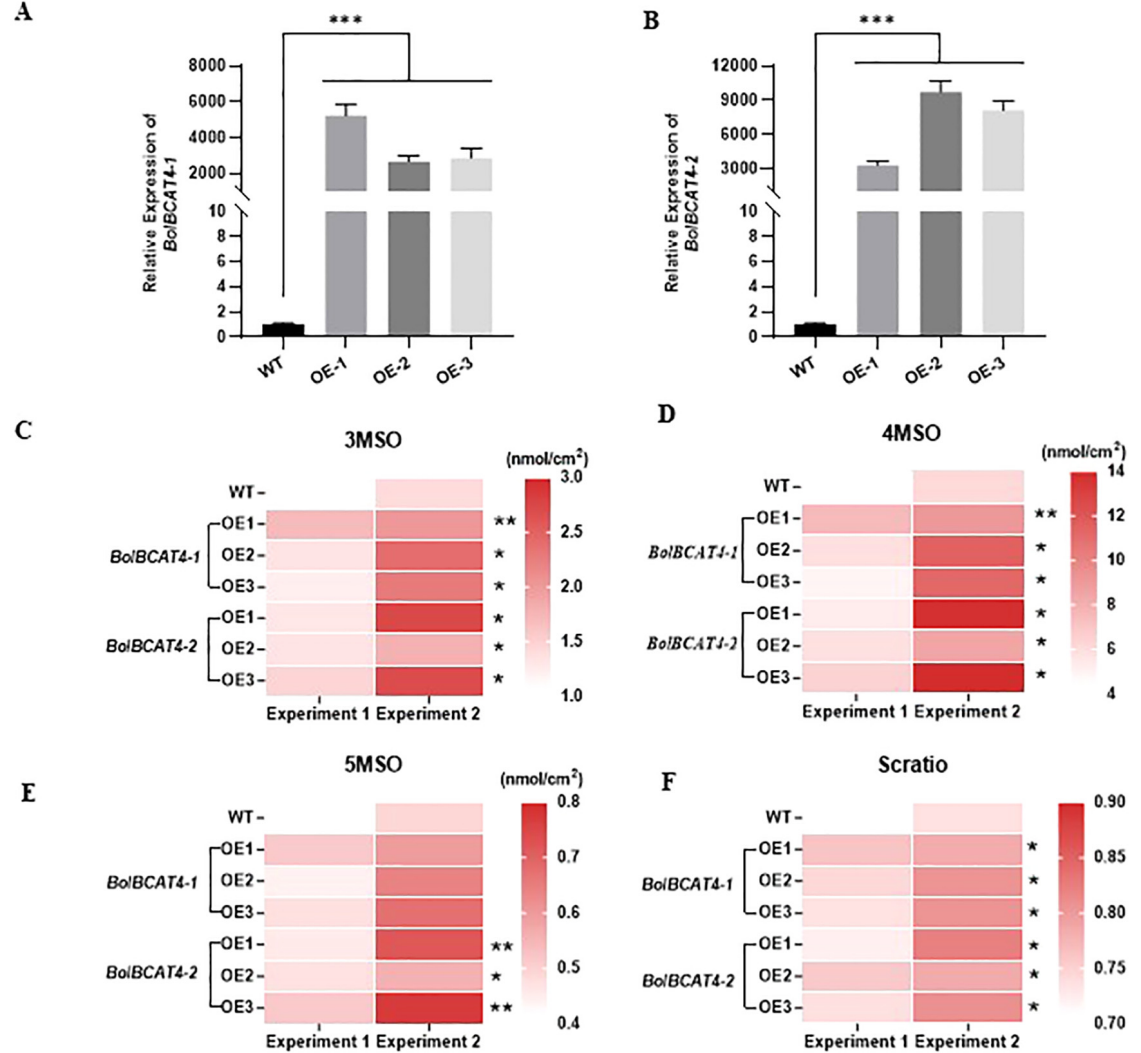
Functional validation of *BolBCAT4-1* and *BolBCAT4-2* as the first set of biosynthetic genes of aliphatic glucosinolates pathway. **(A)** Expression levels of *BolBCAT4-1* in three representative transgenic OE lines of *Arabidopsis*. **(B)** Expression levels of *BolBCAT4-2* in three representative transgenic OE lines of *Arabidopsis*. **(C)** The content of 3MSO in the *BolBCAT4-1* and *BolBCAT4-2* OE lines. **(D)** The content of 4MSO in the *BolBCAT4-1* and *BolBCAT4-2* OE lines. **(E)** The content of 5MSO in the *BolBCAT4-1* and *BolBCAT4-2* OE lines. **(F)** The ratio of short-chain glucosinolates in the *BolBCAT4-1* and *BolBCAT4-2* OE lines. The data from two independent experiments were analyzed by ANOVA. *: *p*<0.05; **: *p*<0.01; ***: *p*<0.001.

To experimentally validate the biological functions of *BolBCAT4-1* and *BolBCAT4-2*, we overexpressed *BolBCAT4-1* and *BolBCAT4-2* in ‘Col-0’ of *Arabidopsis*. In all three representative overexpression lines for both *BolBCAT4-1* and *BolBCAT4-2* (**Figures 2A, B**), the contents of 3-methylsulfinylpropyl glucosinolate (3MSO), 4MSO, and the SCratio (the percentage of short-chain glucosinolates) were all significantly increased (**Figures 2C–F**) (**Supplementary Table 2**). As short-chain glucosinolates are the dominant forms of aliphatic glucosinolates in ‘Col-0’, our data showed both *BolBCAT4-1* and *BolBCAT4-2* are biologically functional in converting Met into aliphatic glucosinolates. Interestingly, overexpression of the *BolBCAT4-2* significantly increased the content of 5-methylsulfinylpentyl glucosinolate (5MSO) (**Figure 2E**), while overexpression of the *BolBCAT4-1* did not, indicating *BolBCAT4-1* and *BolBCAT4-2* also have diverse biological functions.

**Figure 3:**
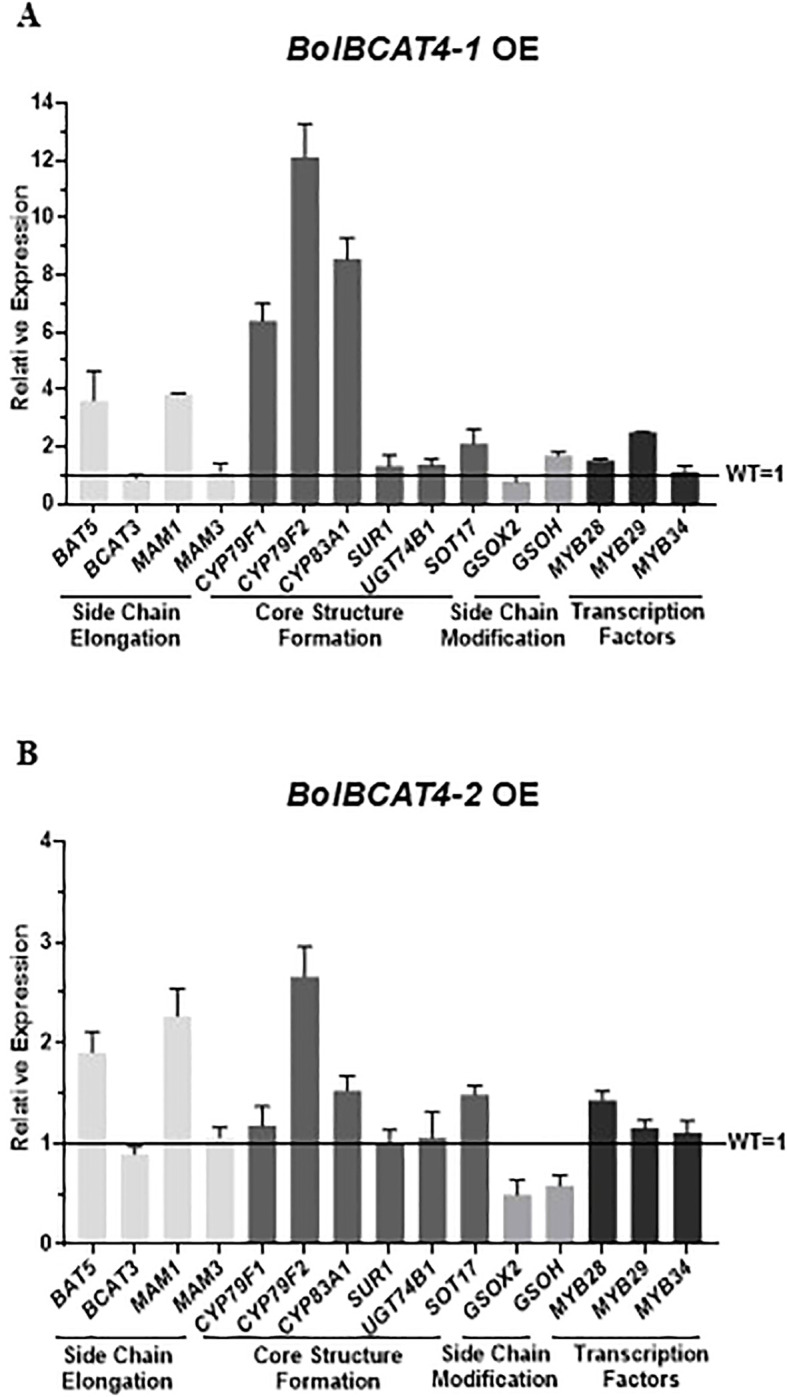
Relative gene expression levels of the aliphatic glucosinolate pathway in the *BolBCAT4s* OE lines. **(A)** Gene expression levels of the aliphatic glucosinolate pathway in the *BolBCAT4-1* OE lines by qPCR. **(B)** Gene expression levels of the aliphatic glucosinolate pathway in the *BolBCAT4-2* OE lines by qPCR.

The expression levels of the genes of the aliphatic glucosinolate pathway in the overexpression of *BolBCAT4* genes showed that multiple biosynthetic genes in aliphatic glucosinolates pathways were also induced due to the overexpression of *BolBCAT4-1*(OE line -1) and *BolBCAT4-2* (OE line -2), especially the genes in the beginning steps of chain elongation and core structure formation (**Figure 3A, B**), which was likely due to the feedback regulatory mechanisms responding to the high accumulation of intermediate metabolites downstream of Met in the aliphatic glucosinolate pathway.

### 2.2 The screening of the upstream regulators of *BolBCAT4* genes

As both *BolBCAT4-1* and *BolBCAT4-2* were biologically functional and, furthermore, they also showed diverse roles in controlling glucosinolate profiling in transgenic plants, we conducted cis-element analysis for 2 kb regions upstream of the *BolBCAT4-1* and *BolBCAT4-2* genes. Large numbers of cis-elements were predicted and identified as potential binding sites for transcriptional regulators (**Supplementary Table 3** and **Supplementary Figure 1**), supporting the importance of the transcriptional regulation of these two genes.

**Figure 4:**
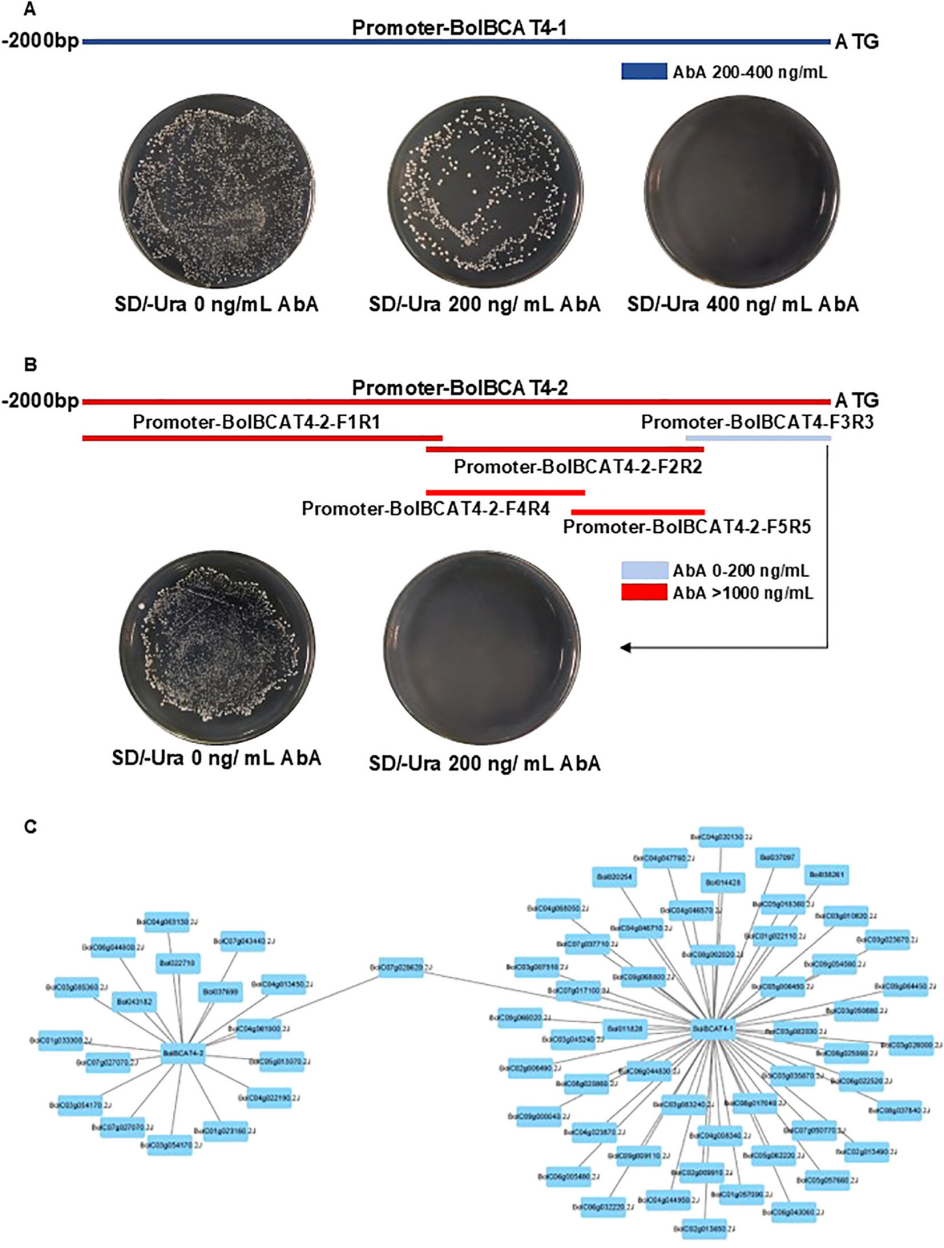
The screening of regulators of *BolBCAT4-1* and *BolBCAT4-2.* **(A)** The cloning and auto-activation test of the 2 kb region of the *BolBCAT4-1* promoter. **(B)** The cloning of multiple truncation forms, and auto activation test of the *BolBCAT4-2* promoter regions. **(C)** The candidate upstream regulators of *BolBCAT4-1* and *BolBCAT4-2* by Cytoscape.

We then used the cabbage cDNA library of our lab (**Supplementary Table 4**) (Bai et al., 2024) to conduct a yeast one-hybrid assay to screen the upstream regulators for the promoters of both *BolBCAT4-1* and *BolBCAT4-2*. For *BolBCAT4-1*, the 2 kb region upstream of the start codon was cloned, confirmed with little self-activation, and used for the following yeast one-hybrid screening (**Figure 4A**). For *BolBCAT4-2*, strong self-activations were found for both the 2 kb promoter region upstream of the start codon, and also for multiple truncation forms of the promoter region. The 426 bp region upstream of the start codon of the promoter of *BolBCAT4-2* was finally used for the yeast one-hybrid (**Figure 4B**, **Supplementary Table 4**). The contrasting self-activation results supported our above findings of the different transcriptional regulations of *BolBCAT4-1* and *BolBCAT4-2*.

Using yeast one-hybrid screening, we identified 57 candidate regulators of *BolBCAT4-1* and 18 regulators of *BolBCAT4-2* (**Figure 4C**, **Supplementary Table 5** and **Supplementary Figure 2**), with one of the candidate regulators, BolC07g028620.2J, binding the promoters of both *BolBCAT4-1* and *BolBCAT4-2*. The identification of these candidate upstream regulators of *BolBCAT4* genes provided valuable resources and research clues for the following functional validation study.

### 2.3 BolMYB3R binds the promoters of *BolBCAT4-1* and *BolBCAT4-2*

**Figure 5:**
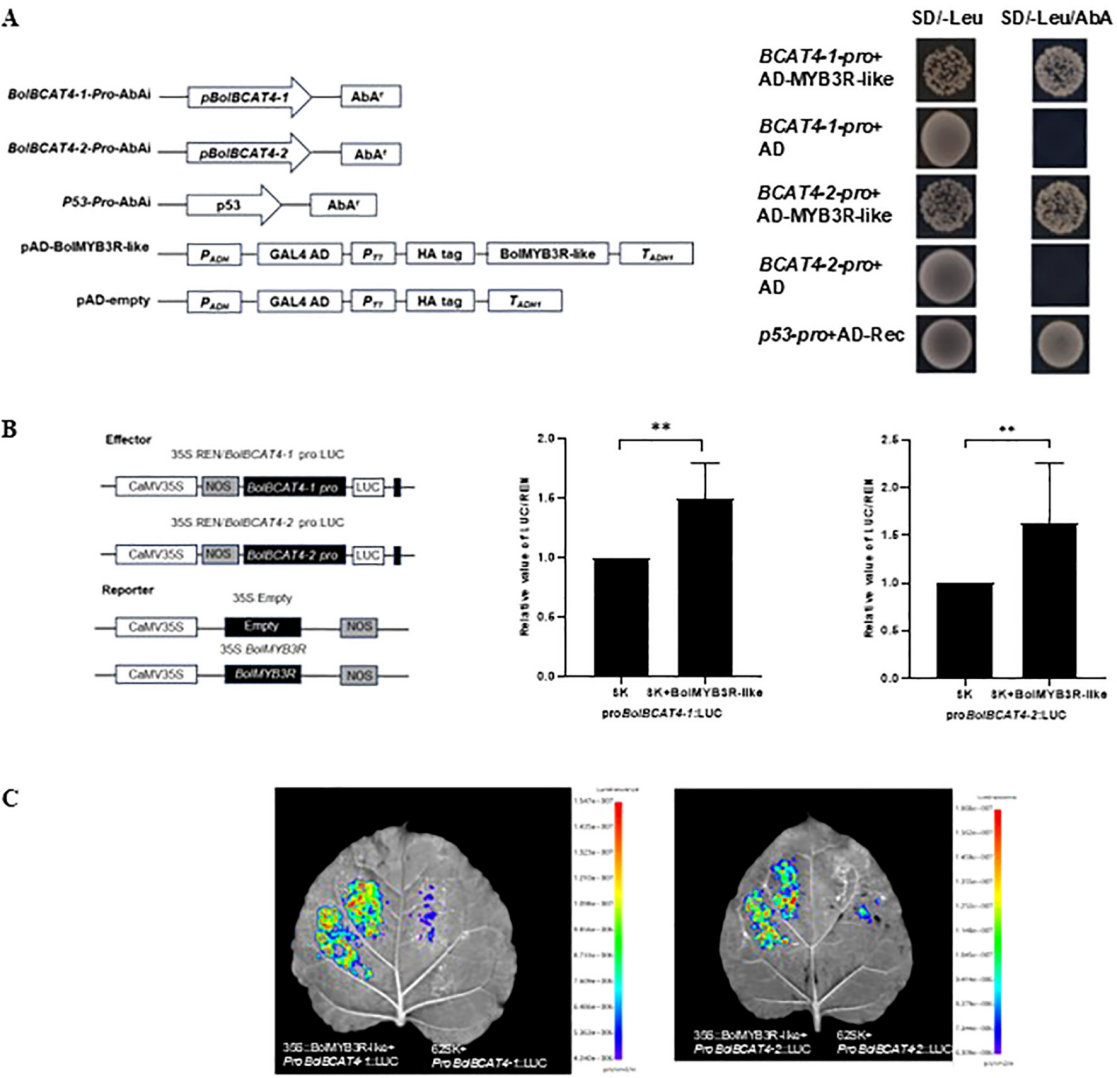
*BolMYB3R* binds the promoters of *BolBCAT4-1* and *BolBCAT4-2*. **(A)** Y1H assays verified that BolMYB3R directly binds the *BolBCAT4s* promoter. **(B)** Dual-luciferase (LUC) assays verified that BolMYB3R increased *BolBCAT4s* promoter activity. The promoter activity is presented by the LUC/REN ratio. **: *p*<0.01. **(C)** Transient expression in tobacco leaves by LUC assays verified that BolMYB3R increased *BolBCAT4s* promoter activity.

Among all the above candidate upstream regulators, we first focused on a MYB-like transcript factor as it belongs to a novel member of the MYB TF family, among which many of the key regulators were previously identified in transcriptionally regulating aliphatic glucosinolate biosynthesis. We named it *BolMYB3R* (*BolC04g046570.2J*) in the following sections. We double-confirmed that BolMYB3R could bind the promoters of both *BolBCAT4-1* and *BolBCAT4-2* using the point-to-point of the yeast one-hybrid assay (**Figures 5A, B**), and also using the LUC assay (**Figure 5C**), supporting BolMYB3R as an upstream regulator of cabbage *BolBCAT4* genes.

### 2.4 The functional validation of BolMYB3R’s roles in modulating glucosinolate biosynthesis

**Figure 6:**
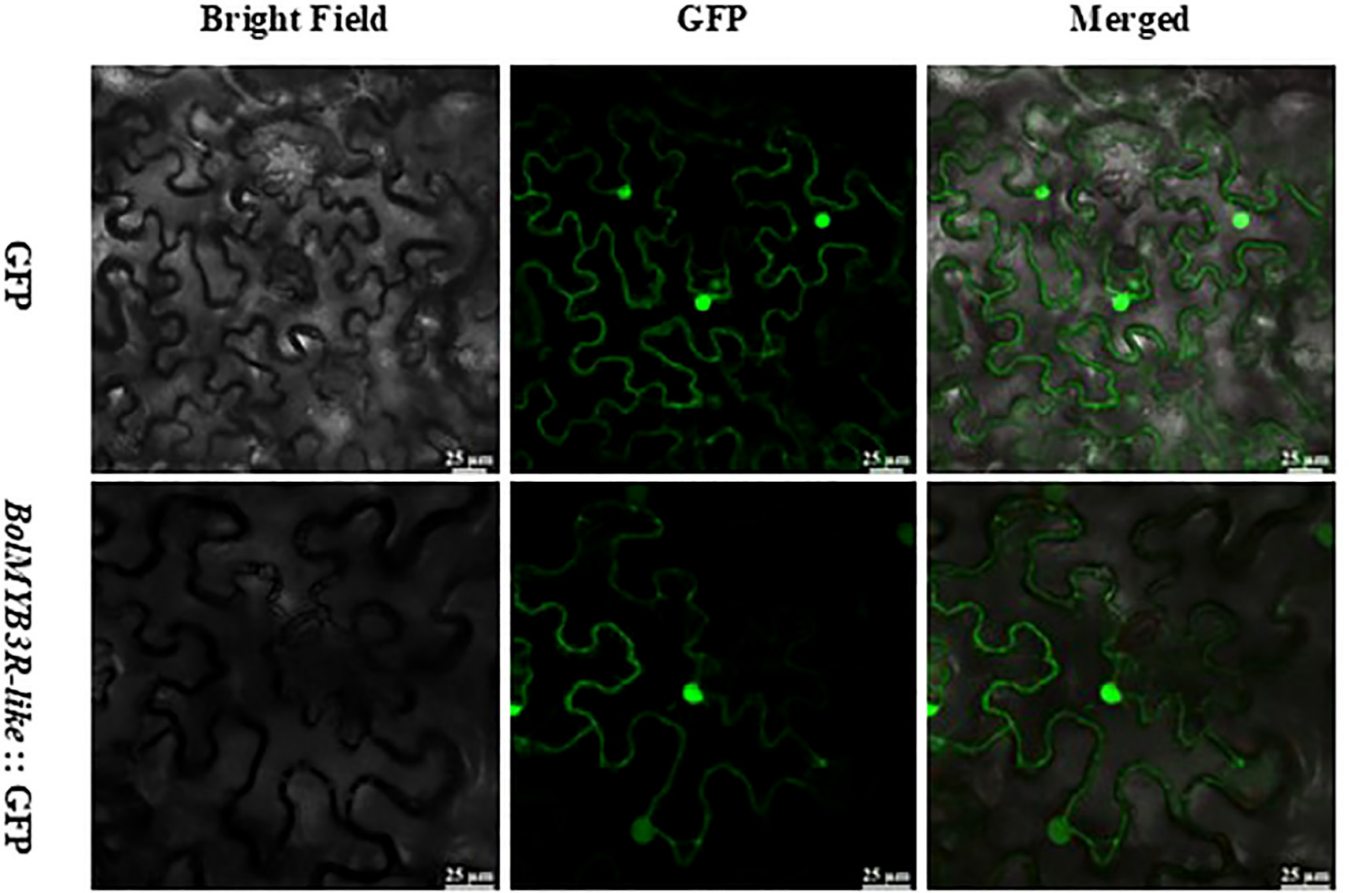
BolMYB3R’s nucleus subcellular location. Scale bar=25μm.

To functionally validate the regulatory mechanisms of BolMYB3R, we first studied its subcellular localization and showed that BolMYB3R was predominantly localized in the nucleus (**Figure 6**), supporting its role as a transcriptional regulator.

**Figure 7:**
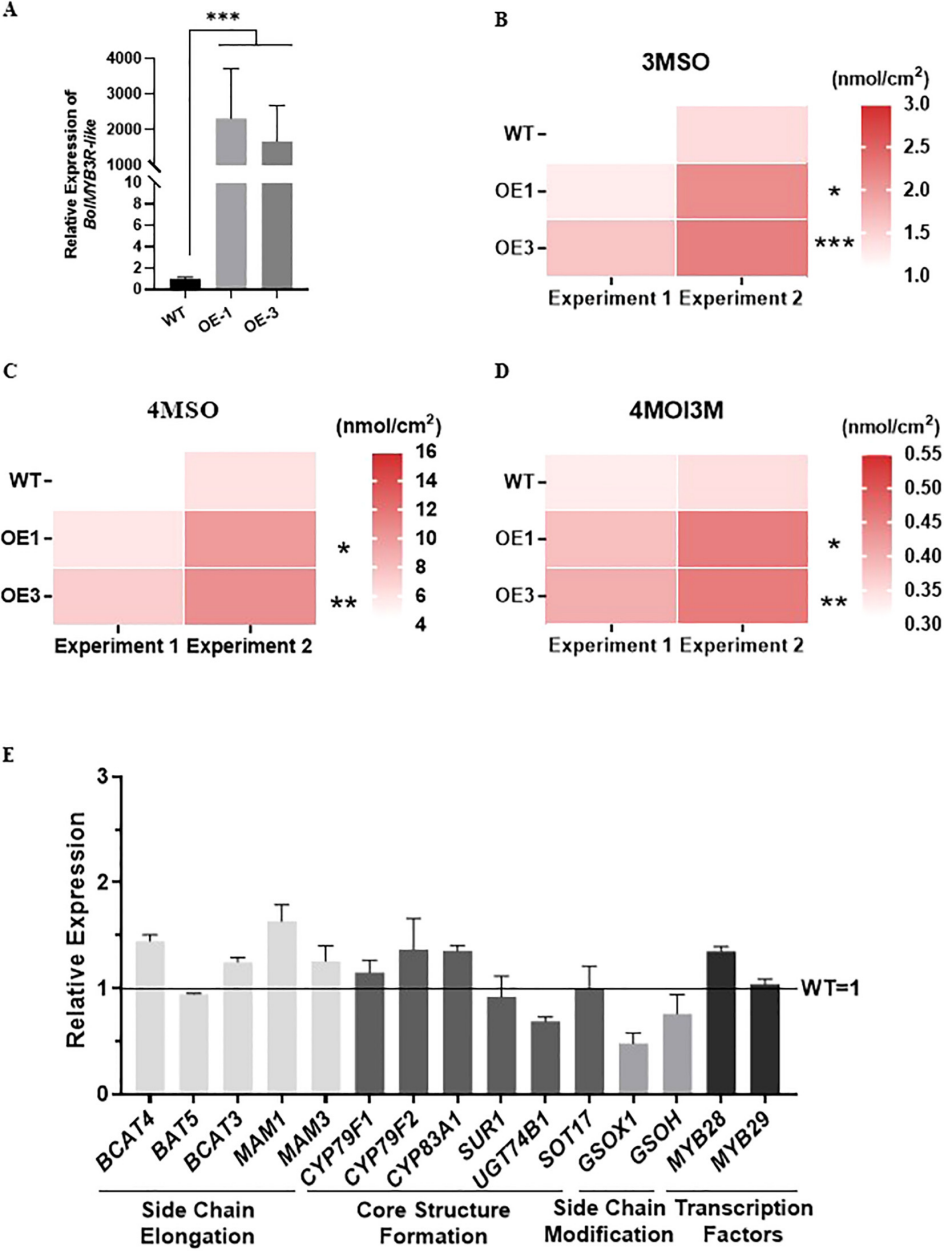
Overexpression of *BolMYB3R* increases short-chain aliphatic glucosinolates. **(A)** Relative expression levels of the *BolMYB3R* OE lines. **(B)** The content of 3MSO in the *BolMYB3R* OE lines. **(C)** The content of 4MSO in the *BolMYB3R* OE lines. **(D)** The content of 4MOI3M in the *BolMYB3R* OE lines. **(E)** Expression levels of the aliphatic glucosinolate metabolism pathway genes in the *BolMYB3R* transgenic *Arabidopsis* line. *: *p*<0.05; **: *p*<0.01; ***: *p*<0.001.

We then overexpressed the *BolMYB3R* in ‘Col-0’ of *Arabidopsis* (**Supplementary Figure 3**), and systematically studied the glucosinolate contents in the two independent overexpression lines. In both of these overexpression lines, the contents of short-chain glucosinolates, 3MSO and 4MSO, were significantly induced (**Figures 7A–C**). As the major phenotypes of the overexpression lines of *BolBCAT4-1* and *BolBCAT4-2* were the induction of short-chain glucosinolates (**Figures 2C, D**), our data clearly showed that BolMYB3R acted as an upstream positive regulator of *BolBCAT4* genes. Interestingly, overexpression of *BolMYB3R* also induced the accumulation of 4-methoxy-indol-3-ylmethyl glucosinolate (4MOI3M)(**Figure 7D**), an important indolic glucosinolate, indicating BolMYB3R also acted as an upstream regulator of the indolic glucosinolate pathway. Most of the expression of biosynthetic genes in the aliphatic glucosinolates pathway in the overexpression lines was moderately induced, which was in accord with the moderate increase in short-chain glucosinolates in the overexpression lines (OE line -1) of *BolMYB3R* (**Figure 7E**).

### 2.5 The functional validation of BolbHLH153, BolMED4, and BolERF74 as upstream regulators of *BolBCAT4* genes

As dozens of candidate upstream regulators of *BolBCAT4* genes were identified in our study (**Figure 4C**, **Supplementary Table 5**), we further selected and functionally validated three more candidate transcriptional regulators. BolbHLH153(*BolC08g002020.2J*) and BolERF74(*BolC03g085360.2J*) were selected because transcriptional factions in the bHLH and ERF families were among the top validated transcriptional regulators of the glucosinolate pathway in our previous study in model plants (Chen et al., 2024; Li et al., 2014a). BolMED4 (*Bol010083*) was selected because few MED regulators have been studied in the transcriptional regulation of aliphatic glucosinolates.

**Figure 8:**
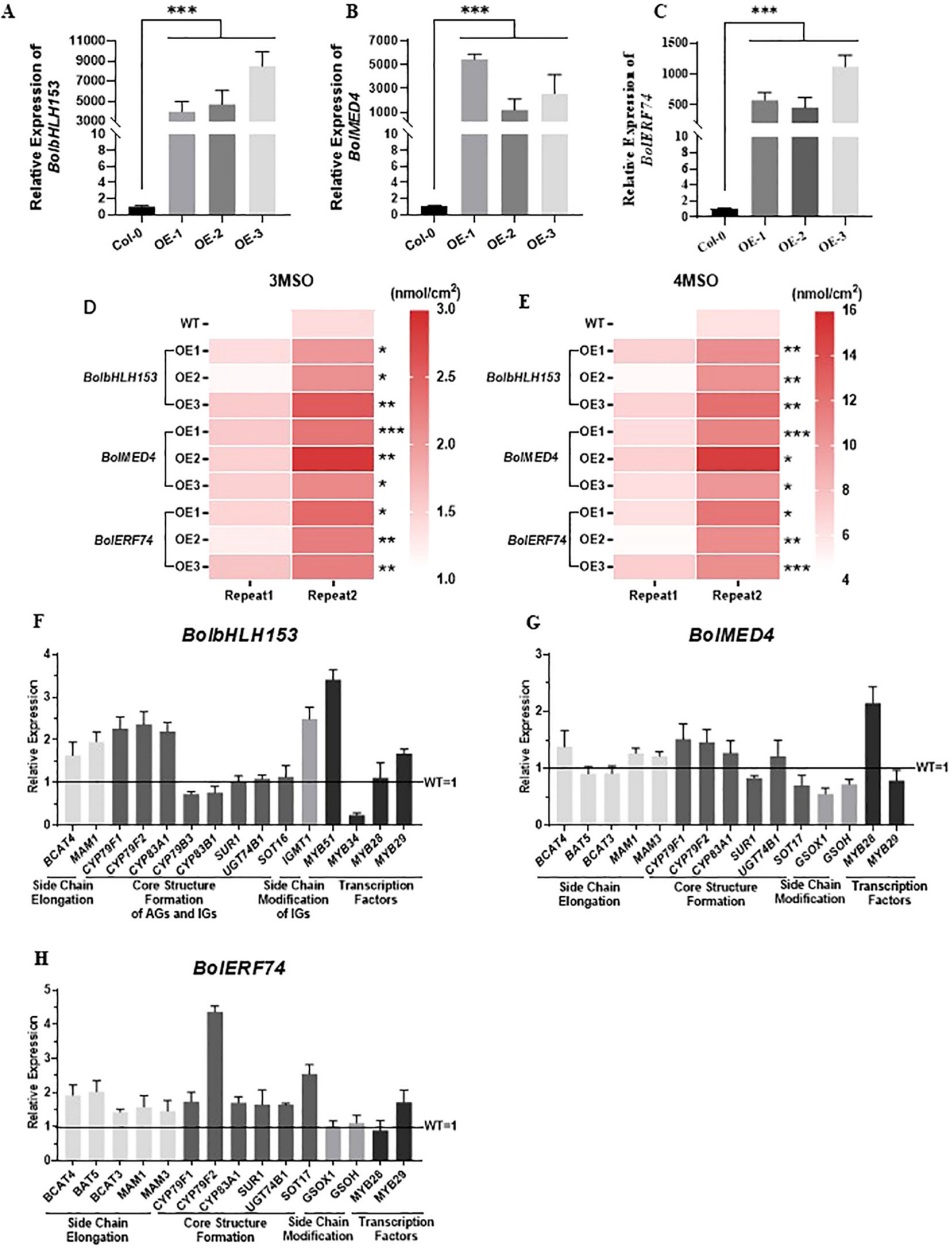
Overexpression of *BolbHLH153*, *BolMED4*, and *BolERF74* increases short-chain aliphatic glucosinolates. **(A–C)** Expression levels of *BolbHLH153*, *BolMED4*, and *BolERF74* in transgenic *Arabidopsis* respectively; ***:*p*<0.001. **(D, E)** Analysis of 3MSO and 4MSO content in *BolbHLH153*, *BolMED4 and BolERF74* overexpression lines; *: *p*<0.05; **: *p*<0.01; ***: *p*<0.001. **(F–H)** Expression levels of the aliphatic glucosinolate metabolism pathway genes in *BolbHLH153*, *BolMED4*, and *BolERF74* transgenic *Arabidopsis* lines.

We generated three independent overexpression lines for each of the three candidate upstream regulators, BolbHLH153, BolMED4, and BolERF74, in Col-0 of *Arabidopsis* (**Figures 8A–C** and **Supplementary Figure 3**). Importantly and consistently, overexpression of *BolbHLH153*, *BolMED4*, and *BolERF74* increased the short-chain glucosinolates, including 3MSO and 4MSO, in all the tested lines (**Figures 8D, E**). The expression analysis of the overexpression lines of *BolbHLH153*, *BolMED4*, and *BolERF74* showed different patterns in these overexpression lines: 1) BolbHLH153 effectively induced the expression of most of the genes in the side chain elongation step and early steps in the core structural formation (**Figure 8F**) (OE line -3); 2) BolMED4 induced the accumulation of short chain glucosinolates mainly through inducing the expression of the key regulator MYB28, indicating hierarchical transcriptional regulatory mechanisms (**Figure 8G**) (OE line -1); 3) BolERF74 strongly induced the expression of early steps of core structural formation, especially CYP79F2 (**Figure 8H**) (OE line -3). In summary, together with the BolMYB3R, we screened, identified, and functionally validated four important positive upstream regulators of *BolBCAT4* genes.

## 3 Discussion

### 3.1 The importance of concerted regulation in the production of aliphatic glucosinolates

In this study, we functionally validated *BolBCAT4-1* and *BolBCAT4-2* and identified and validated four upstream regulators of the *BolBCAT4* genes. One of the key research questions of our study was whether upstream regulators of *BolBCAT4* genes could significantly induce the accumulation of aliphatic glucosinolates. Our data showed both promising data and the complexity of inducing a lengthy secondary metabolism pathway, such as the aliphatic glucosinolates pathway, with multiple biosynthetic steps. 1) On the one hand, overexpression of *BolBCAT4-1* and *BolBCAT4-2*, and the overexpression of four upstream regulators, BolMYB3R, BolbHLH153, BolMED4, and BolERF74, significantly and consistently increased the dominant forms of aliphatic glucosinolates in ‘Col-0’, 3MSO and 4MSO. These findings supported our research hypothesis and validated our major findings. 2) On the other hand, the effects in these overexpression lines were relatively small in both the overexpression line of the *BolBCAT4* genes and the four positive regulators. These could result from the effects of small to moderate induced expression of the genes, and more importantly, it is likely that only coordinated high expression of the genes in the whole pathways would effectively make the final products of lengthy pathways be effectively over-accumulated. It is also likely that only very few key regulators, including MYB28, MYB29, and MYC2, could strongly induce the accumulation of aliphatic glucosinolates, and the rest of the upstream regulators likely mainly work to connect the glucosinolate pathway with other biological processes.

### 3.2 BolMED4 and BolERF74 might regulate the glucosinolate pathway by both direct and indirect modes

**Figure 9:**
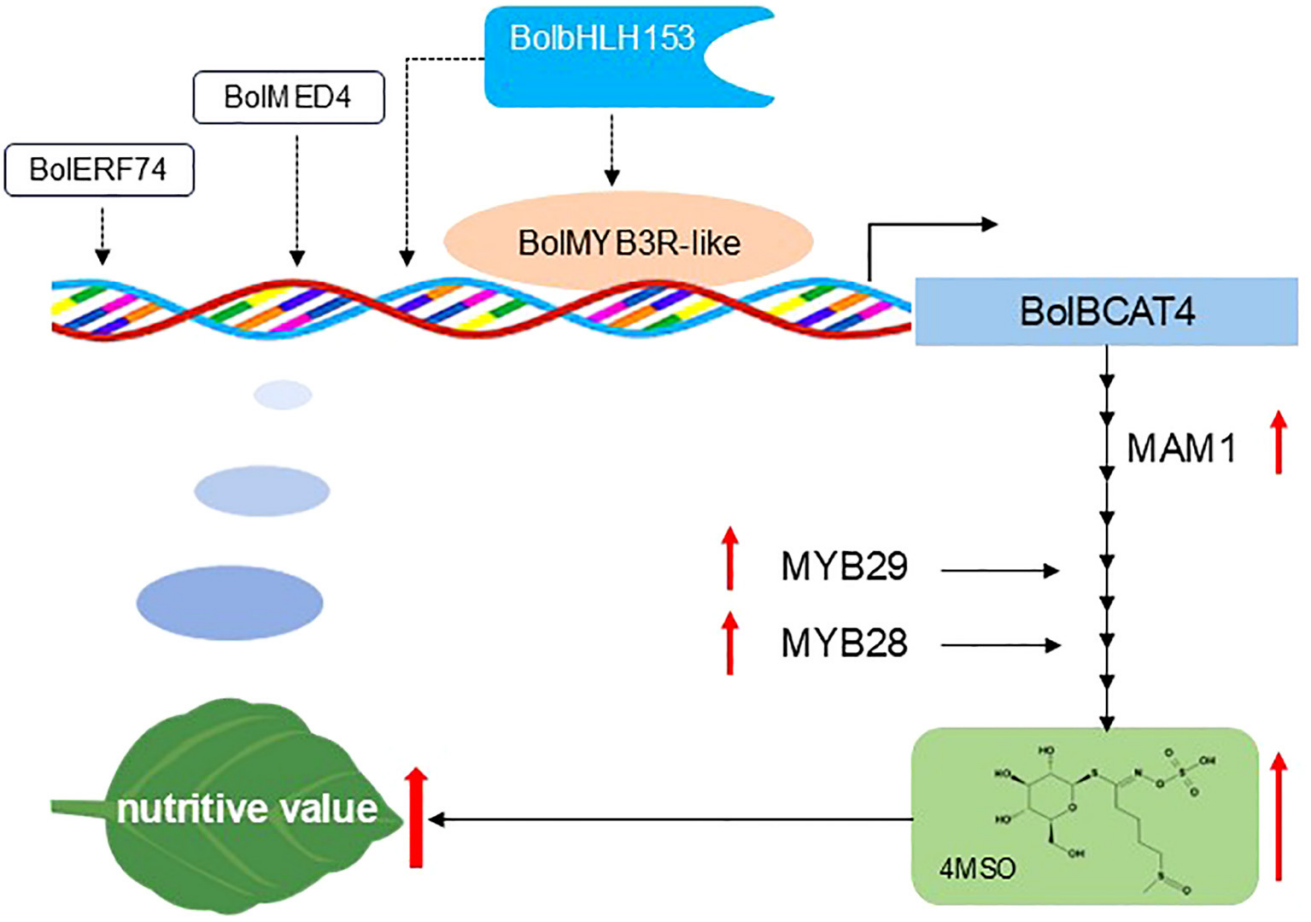
A proposed model explaining how BolMYB3R regulates glucosinolate biosynthesis in *Arabidopsis.* In this model, BolMYB3R directly bound the promoters of *BolBCAT4-1* and *BolBCAT4-2* and induced its expression, increasing the content of short-chain glucosinolates, including 4MSO, as shown in the solid lines. BolERF74, BolMED4, and BolbHLH153 may regulate the expression of *BolBCAT4* genes in an indirect way by forming protein regulatory complexes to regulate the biosynthesis of aliphatic glucosinolate and are indicated by dotted lines.

MED4 has been found to be a mediator protein that encodes a subunit of the plant intermediary complex that interacts with RNA polymerase II to regulate gene expression (Li et al., 2014b; Ma et al., 2023); while ERF74, a member of the AP2/ERF family, is involved in plant responses to hypoxia, oxidation, and osmosis stresses by regulating the expression of specific genes (Kunkowskaa et al., 2023; Munemasa et al., 2013; Xie et al., 2019). In this study, overexpression of *BolMED4* and *BolERF74* increased the content of short-chain aliphatic glucosinolates, indicating BolMED4 may participate in the transcription process through the intermediary complex, affecting the action of RNA polymerase and thus affecting the expression of genes in the aliphatic glucosinolate pathway. ERF74 may regulate the aliphatic glucosinolate pathway by regulating the production and balance of reactive oxygen species when stomatal closure and programmed cell death are induced by isothiocyanate (ITC), which is closely related to the binding of glutathione and reactive oxygen species in wounded plant tissues (Andersen et al., 2013; Andersson et al., 2015) (**Figure 9**).

### 3.3 Positive regulator vs negative regulator

One of the key characteristics of upstream regulators of plant secondary metabolite pathways is whether they are positive regulators or negative regulators, which is particularly important for translational application studies of plant secondary metabolites with high nutritional value or therapeutic value, including aliphatic glucosinolates. Before 2014, almost all the published transcriptional regulators in glucosinolate pathways were positive regulators, while starting from 2014, we identified and found large numbers of negative regulators, or TFs with negative regulatory effects in specific conditions over the past decades (Chen et al., 2024; Li et al., 2014a; Tang et al., 2021). Importantly, in the largest functional validation study of transcriptional regulators in the plant secondary metabolism pathway thus far, we identified an almost balanced distribution of regulators with positive and negative effects for the major aliphatic glucosinolates, short-chain glucosinolates, in model plants (Chen et al., 2024). Interestingly, all four selected transcriptional regulators of *BolBCAT4* genes in our current study were positive regulators, and their effects were also highly consistent in inducing short-chain glucosinolate accumulation. Considering that *BCAT4* is the first gene in the aliphatic glucosinolate pathway, we hypothesized that *Brassica* plants maintain the home keeping and low level of aliphatic glucosinolates, and it was under an internal signal and external stimulus that upstream regulators positively regulate the biosynthesis of aliphatic glucosinolates. It is worth testing the above hypothesis by validating more candidate regulators of *BCAT4* in cabbage and other *Brassica* vegetables, as well as upstream regulators of *CYP79F1/2*, the genes in the first steps in core structural formation. The validated regulators of *BCAT4* are likely to be good target genes for translational application as they might consistently induce aliphatic glucosinolates in diverse growth conditions if the above hypothesis holds true.

### 3.4 Breeding targets for enhancing nutritional quality in cabbage

*Brassica* vegetables play key roles in providing nutrients in human society, especially for the large population with low income in developing countries, and glucosinolates are key components in shaping the nutritional value of *Brassica* vegetables, with 4MSO as one of the best-known functional nutrients. Numerous reports have shown that 4MSO has significant anti-cancer effects (Liou et al., 2020), together with beneficial effects for human health including maintaining intestinal health, and anti-inflammatory and antioxidant properties (Barnabei et al., 2021; Dana and Alejandro, 2022; Seeburger et al., 2023). In our current study, we found that the overexpression of *BolBCAT4* and the four upstream regulators consistently induced the accumulation of 4MSO. Interestingly, the inducing of 4MSO mainly resulted from the increasing ratio of short-chain aliphatic glucosinolates. As four carbon is the dominant form of Met-derived aliphatic glucosinolates in ‘Col-0’, while three carbon is the dominant form in cabbage, it is important to validate whether the phenotypic effects of both *BolBCAT4* genes and the four upstream regulators in our study are also the same in cabbage. If so, both *BolBCAT4* and the four selected transcriptional factors in our study are likely to be valuable breeding targets for increasing the accumulation of 4MSO and enhancing the nutritional quality of cabbage (**Figure 9**).

## 4 Conclusions

*BolBCAT4-1* and *BolBCAT4-2*, the first set of biosynthetic genes of methionine-derived aliphatic glucosinolates in cabbage, were both experimentally confirmed as functional genes. Large numbers of candidate upstream regulators of *BolBCAT4-1* and *BolBCAT4-2* genes in cabbage were screened, and identified; furthermore, four of them were selected and functionally validated as positive regulators, with highly consistent effects of inducing the content of short-chain aliphatic glucosinolates in transgenic plants, including glucoraphanin. Both *BolBCAT4* genes and the four upstream regulators are promising target breeding genes for enhancing nutritional quality in cabbage. Additionally, future efforts in the systemic screening and validating of upstream regulators of biosynthetic genes in the glucosinolate pathway in *Brassica* vegetables will pave the way for achieving the goal of breeding novel cultivars with higher nutritional quality.

## 5 Materials and methods

### 5.1 Plant materials and the conditions for cultivation

*Arabidopsis* (wild type, Columbia-0), cabbage (*Brassica oleracea* var. *capitata* L) line 02–12 (Liu et al., 2014), and tobacco (*Nicotiana benthamiana*) were used in this study. The cabbage and tobacco were seeded into a mixture of matrix, vermiculite, and perlite with a ratio of 1:1:1, and cultivated in a growth chamber at 25 °C, with a light intensity of 125 µmol·m^−2^·s^−1^ and light cycle of 16 h of light/8 h of darkness. *Arabidopsis* seeds were incubated in darkness at 4°C for 48 h to ensure synchronous germination. The seeds were cultured in an incubator at a temperature of 22 °C, with a light intensity of 125 µmol·m^−2^·s^−1^ and light cycle of 16 h of light/8 h of darkness.

### 5.2 Gene cloning and sequence analysis

Sequence information about *BolBCAT4-1* and *BolBCAT4-2* promoters from cabbage line ‘02–12’ were searched for in the Brassica database (http://brassicadb.cn). Promoters of *BolBCAT4-1* and *BolBCAT4-2*, *BolMYB3R*, *BolbHLH153*, *BolMED4*, and *BolERF74* were cloned by PCR amplification from cabbage line ‘02-12’, and subjected to Sanger sequencing, which was performed by Tsingke Biotechnology (Beijing) Co., Ltd. To anticipate cis-acting elements within the *BolBCAT4-1* and *BolBCAT4-2* promoter sequences, the PlantCARE online tool (http://bioinformatics.psb.ugent.be/webtools/plantcare/html/) was employed and these were visualized by TBtools (Chen et al., 2020).

### 5.3 Subcellular localization of BolMYB3R proteins

The coding sequences of *BolMYB3R*, devoid of stop codons, were isolated and incorporated into the GFP fusion vector, pGreen-35S-*BolMYB3R*-GFP. *BolMYB3R*-GFP expression was transiently observed in *N. benthamiana* to confirm the subcellular localization of BolMYB3R.

### 5.4 Yeast one-hybrid assay

The promoter sequence of *BolBCAT4-1* and *BolBCAT4-2* in cabbage line ‘02-12’ spanned 2,000 bp and 426 bp. Subsequently, pAbAi yeast bait vectors were constructed for each of these segments. The yeast one-hybrid (Y1H) library screening assay utilized the Matchmaker^®^ Gold Yeast One-Hybrid Library Screening System from TaKaRa, Japan. To confirm the interaction between BolMYB3R and *BolBCAT4-1*/*BolBCAT4-2*, the coding sequence (CDS) of the *BolMYB3R* genes was inserted into the pGADT7 vector. Additionally, the first fragment of the *BolBCAT4-1* and *BolBCAT4-2* promoter was inserted into the pAbAi vector. In accordance with the Matchmaker^®^ Gold Yeast One-Hybrid Library Screening System instructions from TaKaRa, Japan, the recombinant plasmids were used to transform Y1H Gold yeast cells. Subsequently, screening of the transformed yeast cells was conducted on SD/-Leu medium supplemented with 400 ng/mL and 200 ng/mL aureobasidin A, respectively. The yeast one-hybrid data were visualized using Cytoscape (Shannon et al., 2003).

### 5.5 Dual-luciferase reporter assay

The dual-luciferase assay was utilized to validate the binding of BolMYB3R to the *BolBCAT4s* promoter. To create the reporter construct, a fragment of the *BolBCAT4* promoter was inserted into the pGreenII 0800-LUC vector, and the CDS of *BolMYB3R* was inserted into the pGreenII 62-SK vector to form the effector constructs and subsequently transformed them into Agrobacterium train GV3101, and a mixture of *Agrobacterium tumefaciens* carrying the reporter or effector constructs was infiltrated into *Nicotiana benthamiana* leaves. The *Nicotiana benthamiana* plants that were infiltrated were subjected to 12 h of dark treatment, followed by 2 days of 16 h light/8 h darkness. The activity of the promoter was quantified by calculating the ratio of firefly luciferase (LUC) enzyme activity to the internal reference Renilla luciferase (REN) using a multifunctional microplate reader (Tecan, Männedorf, Switzerland). The LUC/REN value in the absence of BolMYB3R was established as 1. Luciferase activities were measured using the PlantView100 *In Vivo* Plant Imaging System (BLT, Guangzhou, China).

### 5.6 Genetic transformation of BolMYB3R

The encoding sequence of *BolMYB3R* was inserted between the *Xba* I and *Kpn* I sites of the pVBG-2307 vector (Ahmed et al., 2012), and pVBG2307-*BolMYB3R* was constructed and expressed in *Arabidopsis* via the *Agrobacterium tumefaciens* line GV3101. The transgenic seeds were screened on Murashige and Skoog (MS) medium with kanamycin and identified by PCR. Then, 3-week-old unbolted seedlings of wild type (WT) and T3 generations of homozygous lines (OE-1, OE-2) were used for the experiments. The standard floral dip method was used for the transformation of *Arabidopsis*.

### 5.7 Glucosinolate extraction and analysis

The collection procedure of plant leaf samples used for GLS was similar to that described previously (Kliebenstein et al., 2001) but appropriately modified to fit the HPLC platform used in this study. Briefly, 2–3 fully mature leaves were removed from each 3-week-old plant, placed in 1,000 µL of 90% (v/v) methanol, and stored at −80 °C before extraction. Samples were broken using a 2.3 mm metal ball bearing in a paint shaker at room temperature and incubated for 1 h at room temperature. The tissues were centrifuged at 2,500 g for 15 min, and the supernatant was subjected to anion exchange chromatography in 2 mL tubes. After methanol and water washing, the columns were incubated with 210 µL sulfatase solution overnight. The desulfo-GLS were eluted and analyzed by HPLC according to a previously described method (Chen et al., 2024).

### 5.8 RNA extraction and qPCR analysis

Total RNA from leaves of cabbage and rosette leaves of *Arabidopsis* were extracted using the RNA prep Pure Plant kit (Tiangen, Beijing, China), and 1 µg of RNA was used for reverse transcription using a HiScript III 1st Strand cDNA Synthesis Kit (+gDNA wiper) (Vazyme, Nanjing, China). Quantitative real-time PCR (qRT-PCR) analysis was performed by QuantStudio ^®^3 (Life Technologies, Carlsbad, CA, USA) using Hieff ^®^ qPCR SYBR Green Master Mix (Low Rox Plus) (YEASEN, Shanghai, China) with three biological replicates and technical replicates. *AtACT2*(*AT3G18780*) was used as controls. The other primers are given in **Supplementary Table 7**. The relative gene expression was calculated using the 2^−ΔΔCT^ method (Livak and Schmittgen, 2001).

### 5.9 Statistical analysis

Statistical analysis was performed using R Core Team, a language and environment for statistical computing (R Foundation for Statistical Computing, Vienna, Austria; URL, https://www.R-project.org). The statistical significance of qPCR data was analyzed using Student’s t-test within R software. All GLS data were analyzed via ANOVA using a general linear model (Brady et al., 2015). The following model was used to test for differences in GLS accumulation in the mutants within each specific gene to the respective wild-type plants grown concurrently:


$$Y_{gr}=\mu +G+E+\varepsilon_{gc}$$

Y_gr_ represents the GLS accumulation in each plant, genotype G represents the overexpression lines, environment E represents each growth chamber, μ represents the mean value, and ε_gc_ represents the error. All data are presented as the means ± SE (standard error).

## Data Availability

The original contributions presented in the study are included in the article/supplementary material. Further inquiries can be directed to the corresponding authors.

## Supplementary material

Supplementary Figure 1 Analysis of cis-acting elements of *BolBCAT4* promoter. **(A)** Analysis of cis-acting elements of *BolBCAT4-1* promoter. **(B)** Analysis of cis-acting elements of *BolBCAT4-2* promoter.

Supplementary Figure 2 PCR identification of Y1H screening positive clones.

Supplementary Figure 3 Screening, PCR identification of transgenic *Arabidopsis*. **(A)** Screening of *BolMYB3R*, *BolbHLH153*, *BolMED4*, and *BolERF74* transgenic *Arabidopsi*s. **(B)** PCR identification of *BolMYB3R*, *BolbHLH153*, *BolMED4*, and *BolERF74* transgenic *Arabidopsis*.

Supplementary Table 1 The candidate upstream regulators of *AtBCAT4.*

Supplementary Table 2 Glucosinolate abbreviations, descriptions, and chemical structures.

Supplementary Table 3 Analysis of cis-acting elements of the promoters of *BolBCAT4-1* and *BolBCAT4-2.*

Supplementary Table 4 RNA sampling information for yeast library construction in cabbage.

Supplementary Table 5 Summary of screening of lowest AbA inhibitory concentrations of *BolBCAT4* homologous gene bait yeast strains.

Supplementary Table 6 List of proteins interacting with *BolBCAT4* homologous genes in the yeast one-hybrid screen.

Supplementary Table 7 Primers used in this study.
