# Supplementary material for: Transcriptional activation of BolBCAT4 genes enhanced aliphatic glucosinolate accumulation in cabbage
Source: Front Plant Sci. 2025 Apr 15;16:1548003. doi: 10.3389/fpls.2025.1548003 (PMC12037493; doi:10.3389/fpls.2025.1548003)
Supplement: Supplementary Figure 1 — Analysis of cis-acting elements of BolBCAT4 promoter. (A) Analysis of cis-acting elements of BolBCAT4-1 promoter. (B) Analysis of cis-acting elements of BolBCAT4-2 promoter. [file DataSheet1.pdf]

Supplementary Material

1 Supplementary Figures and Tables

1. Supplementary Figures

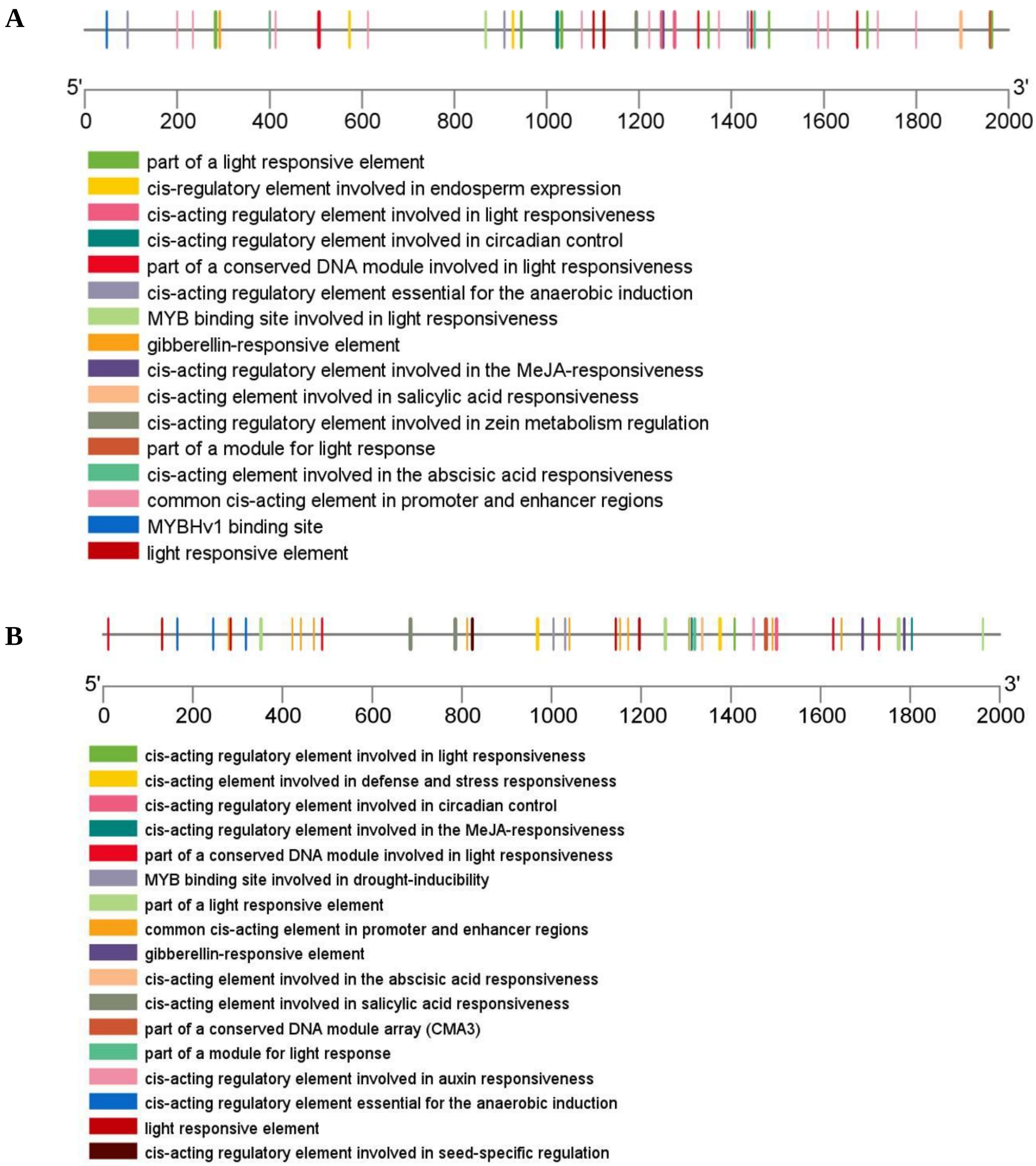

Supplementary Figure 1. Analysis of cis-acting elements of BolBCAT4 promoter. (A) Analysis of cis-acting elements of BolBCAT4-1 promoter. (B) Analysis of cis-acting elements of BolBCAT4-2 promoter.

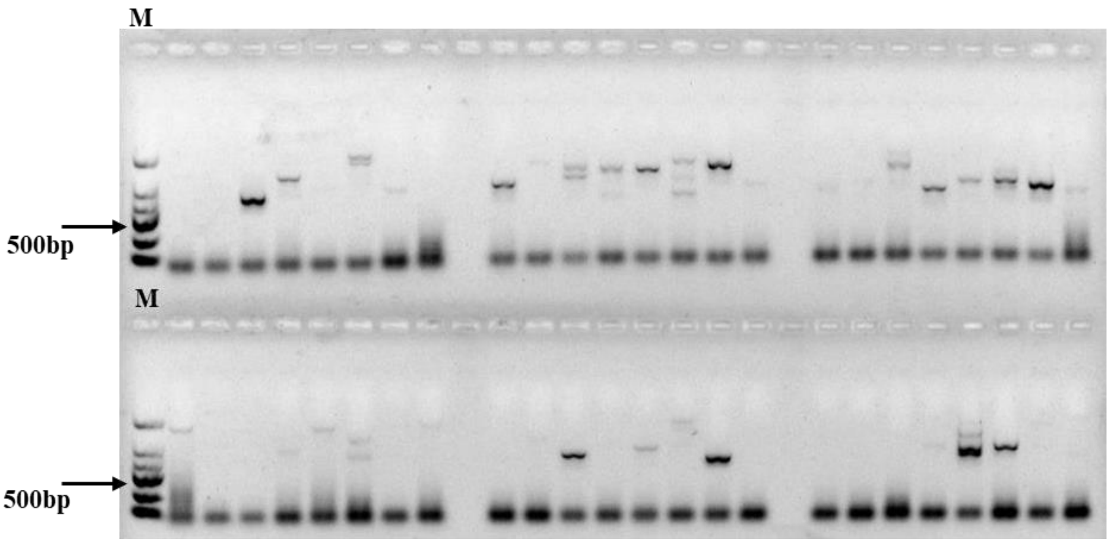

Supplementary Figure 2. PCR identification of Y1H screening positive clones.

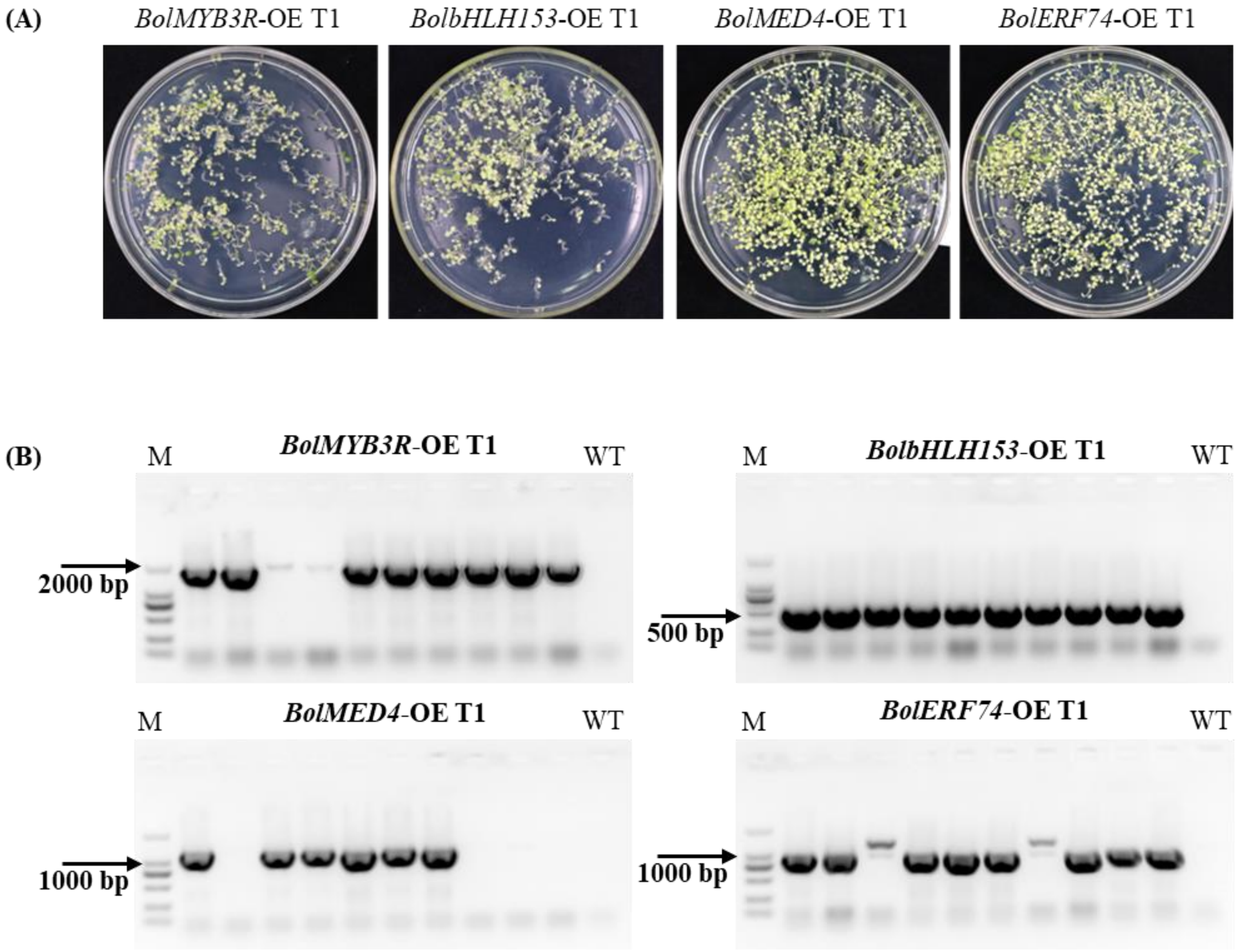

Supplementary Figure 3. Screening, PCR identification of transgenic Arabidopsis. (A) Screening of *BolMYB3R*, *BolbHLH153*, *BolMED4* and *BolERF74* transgenic Arabidopsis. (B) PCR identification of *BolMYB3R*, *BolbHLH153*, *BolMED4* and *BolERF74* transgenic Arabidopsis.

1.2 Supplementary Tables

Supplementary Table 1. The candidate upstream regulators of *AtBCAT4*.

Supplementary Table 2. Glucosinolate abbreviations, descriptions, and chemical structures.

Supplementary Table 3. Analysis of cis-acting elements of the promoters of *BolBCAT4-1* and *BolBCAT4-2.*

Supplementary Table 4. RNA sampling information for yeast library construction in cabbage.

Supplementary Table 5. Summary of screening of lowest AbA inhibitory concentration of *BolBCAT4* homologous genes bait yeast strain.

Supplementary Table 6. List of proteins interacting with *BolBCAT4* homologous genes in the yeast one-hybrid screen.

Supplementary Table 7. Primers used in this study.
